# Questionnaire study to gain an insight into the manufacturing and fitting process of artificial eyes in children: an ocularist perspective

**DOI:** 10.1007/s10792-016-0383-4

**Published:** 2016-10-28

**Authors:** Holly Chinnery, Simon B. N. Thompson, Siamak Noroozi, Bryce Dyer, Karen Rees

**Affiliations:** 1 Psychology Research Centre, Faculty of Science & Technology, Bournemouth University, P104, Poole House, Talbot Campus, Fern Barrow, Poole, Dorset BH12 5BB UK; 2 Clinical Psychology and Neuropsychology, Psychology Research Centre, Faculty of Science & Technology, Bournemouth University, Poole, UK; 3 Design Simulation Research Centre, Faculty of Science and Technology, Department of Design and Engineering, Bournemouth University, Poole, UK; 4 Research & Professional Practice and Principal Academic, Faculty of Science and Technology, Department of Design and Engineering, Bournemouth University, Poole, UK; 5 Public Health/Health Visiting, Faculty of Health and Social Science, Department of Nursing and Clinical Science, Bournemouth University, Poole, UK

**Keywords:** Artificial eyes, Child healthcare, Prosthetic eyes, Healthcare

## Abstract

**Purpose:**

To gain an insight into the manufacturing and fitting of artificial eyes in children and potential improvements to the process.

**Method:**

An online qualitative survey was distributed to 39 ocularists/prosthetists in Europe and Canada. Participants were recruited through purposive sampling, specifically maximum variation sampling from the researcher’s contacts and an online search.

**Results:**

The findings highlighted the current impression technique as being the most difficult yet most important part of the current process for both the ocularist and child patient. Negatively affecting obtaining a good impression, the child patients distress can be reduced by their parents by providing encouragement, reassurance, practicing the insertion and removal of the artificial eye and being matter of fact. Whilst improvements to the current process provided mixed views, the incorporation of current technology was perceived as not being able to meet the requirements to produce aesthetically pleasing artificial eyes.

**Conclusion:**

The current artificial eye process can be seen as an interaction with its success being dependent on the child patient’s acceptance and adjustment which is dependent on the factors associated to the process. Investigation into the needs of the patient and whether technology can improve the process are the next steps in its advancement.

## Introduction

In recent years, technology has been incorporated into healthcare with areas such as biotechnology and medical devices making significant contributions to peoples’ health and wellbeing. Although the use of technology into prosthetic eyes has started to be investigated, the focus has been on orbital prosthesis (whom make up only 1% of prosthetic eye wearers) and not on artificial eyes.

Research into orbital prosthesis development has investigated the use of CT and MRI scans to create an accurate representation of the socket as well as the use of CAD/CAM software and rapid prototyping to design and manufacture the prosthesis [1–5]. Whilst these methods show potential, the specific materials and process requirements need to be addressed in order to create an orbital prosthesis that is functional and aesthetically pleasing [1, 4].

Whilst orbital prostheses contain both polymethyl methacrylate (PMMA) and silicon and are retained by skin adhesives or orbital bone implants, artificial eyes are made from PMMA only and occupy an anophthalmic socket. With different designs and manufacturing criteria, the application of technology to the type of prosthetic will vary, thus what is suitable for one may not be suitable for the other.

Although healthcare has incorporated the psychological wellbeing of the patient into the medical treatment, the focus still largely remains on the cause, such as the disease, trauma or injury, rather than the associated after effects. The loss of an artificial eye can evoke emotional and behavioural responses of the affected individual, their family and their friends all of which may negatively impact the quality of life of the affected individual [6–8]. Exacerbation of the psychosocial difficulties is caused when the reasons behind the loss are not treated with sensitivity and understanding [6] and when the replacement is not done in a timely manner: both of which impact the patient’s acceptance of the artificial eye [7, 9–11]. Despite the role of the patient’s psychological wellbeing in acceptability of the prosthetic, the current care provision still focuses on medical and surgical interventions to treat appearance-related concerns [12].

To further our understanding of a patient’s psychological wellbeing, investigation of the impact the current manufacturing and fitting process has on the patient is needed. Difficulties in the process can affect the patient’s wellbeing resulting in reduced satisfaction with the outcome. Understanding the process and the factors that impede and aid it can lead to the development of better methods (investigating the role of technology) resulting in improved psychological wellbeing.

Those directly involved in the care process, whether that is staff, patients or their family members, are best placed to provide understanding and inform better practice. This is known as a ‘bottom-up’ approach in healthcare service research resulting in increased overall effectiveness and performance.

Healthcare service research commonly uses qualitative methodologies to grasp the meaning, reasons and patterns behind what is being observed [13, 14]. Aspects of healthcare delivery that influence performance and quality of care will be highlighted, setting the path for implementing change [15, 16].

Thus, the aim of the study is to gain insight into the manufacturing and fitting process of artificial eyes in children with a diagnosis of retinoblastoma, from the perspective of ocularists. Specific attention will be paid to the current process, the impact of the process on the patient and the incorporation of technology into the process.

## Method

### Data collection

Surveys are a commonly used method of collecting information about a population of interest. Describing numerical distributions of variables in the population rather than exploring meaning and experiences [17, 18], surveys are typically seen as quantitative in nature rather than qualitative. With the need to use an accessible method of data collection due to the geographical location of participants, a qualitative survey was deemed the most appropriate. The survey was delivered via an online platform to reach a target audience.

### Sampling

As a qualitative study, the sample needs to be appropriate to the research aims and assumptions and provide an adequate amount of data to fully analysis the topic being studied [19]. Braun & Clarke (2006) suggest a sample size of 15–20 for qualitative surveys. Due to the small population size and the likelihood of non-response, all potential participants identified will be included in this study.

The sampling method will be purposive, specifically maximum variation (heterogeneous) sampling. As a non-probability sampling method suiting small populations, a wide range of perspectives from specific characteristics of the population will be attained, allowing for greater insights into the phenomenon being investigated [19].

### Participants

Three factors were taken into consideration: job role, experience and population size. Potential participants needed to be ocularists/prosthetist’s and have experience of fitting artificial eyes in children particularly those with a diagnoses of retinoblastoma. Due to the small population size within the UK and a need to represent the diversity of the phenomenon under study (Jansen 18), the study parameters were extended to include participants in Europe and Canada. The benefits to these territories include Europe having a similar culture and medical system to the UK and Canada as a leading expert in retinoblastoma research, both of which increases the knowledge of the phenomenon. Participants were identified from the researcher’s contacts, conference attendance and from an online search.

### Study design

The questionnaire was developed based on previous literature and discussions with the population group. The questionnaire included questions regarding the current manufacturing and fitting process of artificial eyes in children, the impact of the process upon the child patient, influence of the parents upon the child’s adjustment to the process and the incorporation of technology. Example items include ‘What are the main difficulties you encounter in fitting artificial eyes in children?’, ‘Based on your experience, how do parent’s/guardians contribute to the artificial eye process?’ and ‘What are your thoughts on incorporating technology into the current process?’.

### Ethical approval

Ethical approval for this study was obtained from Bournemouth University Ethics Board. All procedures performed in this study involving human participants were in accordance with the ethical standard of Bournemouth University and with the 1964 Helsinki declaration and its later amendments or comparable ethical standards.

Participation was voluntary and anonymous. No personal information was collected or accessed. Consent for anonymous data to be used was indicated by ticking their agreement to participation before starting the survey. Participation burden was minimised by the use of online surveys allowing the participant to reply in their own time and in their own environments. Respondents derived no personal benefit from taking part in the survey.

### Recruitment

Following identification of potential participants, an email invitation introducing the study and asking for their participation was sent. The email contained an information sheet detailing the purpose of the study, the inclusion criteria, their role, confidentiality and their right to withdraw during and up to one week after completion. Potential participants who meet the inclusion criteria and were happy to take part in the study were asked to use the link contained in the email to participate. On opening the link to the survey, participants were asked to re-read the information sheet and to tick a box conveying an understanding of their rights and agreeing to their participation.

The online survey was conducted using the online platform ‘Survey Monkey’. Initially, participants were given five weeks to complete the survey. After two weeks, an email reminder was sent to all participants. Due to a low response rate, it was decided that the survey invitation would be extended by another two weeks. All participants were informed of the change in timeline. Five days before the end of the survey, a follow-up email was sent to all participants expressing the importance of their contribution to this survey.

### Data analysis

The research approach used often dictates the type of data analysis undertaken. Running the risk of leaving important knowledge hidden in the data, especially when the topic being researched is under-studied, incorporation of both types of analysis will minimise the limitations and maximise the advantages of each approach. Descriptive statistics (means, standard deviations and percentages) will be carried out alongside coding to gain a rounded knowledge of the phenomenon being investigated. Further statistical analysis was not deemed necessary due to the study being qualitative in nature.

## Results

A total of 39 email invitations containing the link to the online survey were sent to ocularists and prosthetists in 14 countries across Europe and Canada. Two participants did not take part in the study: one due to relocation outside of the geographical inclusion criteria, and one to the email address not being recognised. This left a final total sample of 37 participants, or 94.9% of the invited group.

A response rate of 46% or 17 replies was achieved from six countries. The UK and Canada had the highest response rate, (UK: 47%; Canada: 29%), with the number of respondents from these countries being 62%, or eight participants and 50% or five participants, respectively. Spain had a 50% response rate (two participants invited) with the remaining three countries (Belgium, Ireland and Romania) having a 100% response rate (one participant per country invited).

### Sample demographics

71% of respondents were from Europe with the remaining 29% of respondents from Canada. For a breakdown by country, see Table 1. The sample comprised ocularists and prosthetists, a ratio of approximately 70:30. Two of these participants also identified themselves as ‘lead tutors’, one as a dental technician specialising in maxillo-facial prosthesis, and one as an optician and technical orthopaedic. Respondents reported a wide variation in years working as an ocularist/prosthetist ranging from 1 to 5 years, to, 31 years plus: the majority of which being between 11 and 20 years (41%). The variations in years of services did not appear to be country specific.

**Table 1:** Country of service of respondents (%)

Country of service	Respondents (%)
UK	47
Canada	29
Belgium	6
Ireland	6
Romania	6
Spain	6

% Percentage

Respondents reported apprenticeship (50%) and dentistry qualification (29%) as a job requirement with 21% stating no job requirement was necessary. Previous experience includes dentistry (64%), a background in art (9%) and none (27%).

Demographic data showed similarities and variations in the way participants from different countries responded. The sample from Europe and Canada were equivalent in terms of previous experience being dentistry. Furthermore, Canada and 60% of countries from Europe identified themselves as ocularists. Respondents who identified as prosthetists (Spain and 62% of UK respondents) are primarily from services that split the fitting and manufacturing process.

Differences in the demographic data mainly exist between Europe and Canada and are in relation to training and guidelines followed. In comparison to Canada where training to become an ocularist involves attendance to the American Society of Ocularists where formal examinations are undertaken and guidelines followed, there are no formal qualifications to become an ocularist in Europe. Most of the training and guidelines followed are a result of in-house training, teaching skill courses, NHS training (UK only), conferences and sharing experiences with other ocularists.

### Current process of manufacturing and fitting artificial eyes in children

The main indicators for an artificial eye include cancer, trauma and injury. This is followed by infection, painful blind eye and road traffic accidents. The time-span on replacing an artificial eye with a new one varied between respondents whom stated either 1–2, 2–3 or 3–4 years. The need for a change is often dependent on growth, bad fit, loss and psychological adjustment.

Regarding the manufacturing process, all respondents reported using PMMA to manufacture artificial eyes. 78% of respondents reported using the empirical/impression technique in the fitting process: 29% of which uses sedation/examination under anaesthesia. 11% of respondents reported that the technique used is case dependent and a further 11% highlighted the importance of making the appointment fun and using toys and books to achieve a good fit. Various advantages and disadvantages of the current process were highlighted by respondents (see Table 2).

**Table 2:** Advantages and disadvantages of the current fitting process of artificial eyes

Advantages (%)	
Accuracy	20
Nice working environment	20
Consistency	10
Confidence of procedure by parents	10
Multiple fitting procedures	10
Painless	10
Custom made	10
Helps with the child’s wellbeing	10
Disadvantages (%)	
None	50
Lack of time	37.5
Non-corporation of parents	12.5
Unsure	12.5

% Percentage

77% of respondents reported obtaining a good fit (accurate impression, comfort and getting enough pressure for orbital growth), cosmesis (symmetry and position of the iris), understanding parents and the child patients' concerns, setting realistic goals and showing compassion as important factors in fitting artificial eyes in children.

36% of respondents reported that the current process of manufacturing and fitting artificial eyes has remained the same with 9% being unsure whether a change has occurred. All respondents whom stated a change has occurred put it down to factors outside of the design and manufacturing process made such as flexible decision making, knowledge of materials and psychological difficulties, understanding of the anatomy and involving the child in the treatment process.

### Psychological impact of the artificial eye process on the child

75% of respondents reported the fitting process as more distressing for the child than the loss of an eye. However, this is dependent on the age of the child and the reason for the loss. A distressed child may exhibit, fear anger, shyness, stress and distrust which can be exacerbated by pain and psychological adjustment all of which can have a negative impact on the process. However, this is not always the case: 18% of participants reported the child patient experiencing no emotional difficulties.

Creating an impression and the influence/reaction of the parents were found to be factors impeding the process for both the ocularist and the child patient. See Table 3 for influence of parents on the process. Consequently, the process is slowed down, making it difficult to get the best fit, thus affecting the outcome. Other factors that influence the child patient’s distress include the location of the service and repeated visits. Incorporating parents into the process can reassure/distract the child patient as can the use to foys/pictures and other staff members. Managing expectations, making time, involving the child in the process where they can ask questions and using age appropriate language are other key factors in overcoming the difficulties encountered. Respondents reported that the child’s distress is ultimately reduced when they become accustomed to the process whereby they are able to communicate and understand what is happening. Whilst this can vary among children, respondents reported that it can occur between the ages of 2 and 8 years.

**Table 3:** Parents contribution to the fitting process

Positives	Negatives
Relaxed/calm	Too soft
Being matter of fact	Coddle their child
Reassuring	Transmit stress/fear
Encouraging	Show fear Do not practice inserting and removing the artificial eye

Distress on behalf of the child patient and the parent is largely managed by the ocularist. 65% of respondents reported not operating a screening process or protocol in identifying and supporting these patients. Respondents from Canada reported that patients indicating a need for support services are referred back to the physician whom will make the referral. In Europe, referrals are made on an individual basis mainly to counselling or peer support services: this is done by the ocularist.

### Incorporation of technology into the artificial eye process

Respondents thoughts on incorporating technology into the manufacturing and fitting process were mixed with 67% of respondents responding positively. Suggested benefits of using advancements in technology include evolving the field from the current process which is seen as outdated, improved cosmesis; eliminating/lowering fear in the patient conveying confidence to the patient and, where applicable, their parents/family. Reasons for keeping the process the same include the process being seen as a skill and craft that needs to be preserved, the current method being seen as sufficient and current technology being unable to produce the results required (for example, it is unable to show soft-tissue displacement therefore cannot produce a good fit).

Although respondents reported that technological advancements can help produce better implants and improved cosmoses, 57% were unable to suggest ways that technology could enable this. Two respondents reported currently working on developing artificial eyes. This included creating a light reactive pupil prosthesis which will improve general realism and assist in better confidence.

Developments in other fields were acknowledged whilst highlighting its limits in terms of artificial eye development. Whilst 3D printing and the printed iris pattern were highlighted as having the potential to affect future change in the manufacturing of artificial eyes, emphasis was placed on the need to refine techniques and develop the appropriate equipment to move the practice forward. Being a small market where research and development of new ideas are limited due to a lack of funding is also seen as affecting the ability to advance the process.

Respondents also reported the manufacturing and fitting guidelines as having an impact on technological development. With varying regulations in Europe, respondents highlighted the need to create standards of care across the board that address various issues such as training and certification. Once seen as a field in its own right, funding can be achieved where development of the process can begin.

## Discussion

The aim of this qualitative study was to gain an insight into the manufacturing and fitting process of artificial eyes in children diagnosed with retinoblastoma from the perspective of ocularists. The findings from the analysis highlighted difficulties in the process, suggestions for how they can be resolved and whether technology can be incorporated into the current process. The impression technique was identified as being the most important, yet also the most difficult aspect of the current process for both the ocularist and the child patient. Negatively affecting obtaining a good impression, the child patients distress can be reduced by their parents by providing encouragement, reassurance, practicing the insertion and removal of the artificial eye and being matter of fact.

Whilst improvements to the current process provided mixed views, the incorporation of current technology was perceived as not being able to meet the requirements to produce aesthetically pleasing artificial eyes. These findings enhance understanding of the steps needed to improve aspects related to artificial eyes.

Early loss of an eye leads to contraction of tissues around the socket, reduced socket size and depth, decreased residual muscles and shortening of the eyelid rims resulting in asymmetry [20–22]. Consequently, periodic enlargement is required to aid the normal development of the lids and the soft-tissue lining the orbital bone [20, 22–25]. Variations in how often children require new artificial eyes were highlighted in this study, ranging from 1to 4 years with 2 years being the average. Rather than provide a time-span to the change of an artificial eye, Raizada et al. [23] and Shaikh et al. [24] reported that a change is indicated when the prosthesis rotates within the socket, lose fit, decentration of the cornea and discolouration of the prosthesis.

The results of this study showed that the fitting process can be distressing for the child as well as the loss of an eye. Previous literature highlighted a relationship between the loss of an eye and anxiety, depression and stress [24]. The same psychological difficulties were also found by this study regarding the current manufacturing and fitting process.

The psychosocial and cosmetic disorders experienced by the child patient can make behaviour management difficult [20]. In keeping with the literature, this study highlighted trust, patience, reassurance and understanding by the ocularist as important aspects in managing the child patients distress [20].

The incorporation of technology has started to be investigated in regard to orbital prosthesis, yet with different requirements, attention needs to be paid to technological development regarding artificial eyes. In keeping with the literature, this study highlighted the potential effectiveness of CAD/CAM software and rapid prototyping, yet refinement of the techniques is needed to ensure correct soft-tissue placement, good fit and cosmesis [1–5].

### Strength and limitations

Utilising both a quantitative and qualitative approach is ideal when samples are too small to provide scientific valid sample sizes yet are too important to ignore and when topics are critical but not yet sufficiently well understood to assign meaningful numbered responses. This approach can reduce the biases and limitations of both qualitative and quantitative approaches. Combining both questions types allowed a variety of data to be gathered, which either question type could not attain on its own.

This study benefited from recruiting ocularists across Europe and Canada as it highlighted differences and similarities, where learning points can be made. The two population areas were equivalent in the current process of manufacturing and fitting artificial eyes; however, differences occurred in terms of training, qualifications and guidelines followed. These findings can help to develop clarification and uniformity of procedures used.

Literature concerning response rates to online surveys are inconsistent. Converse et al. [25] reported a response rate for electronic surveys at 25–30%. However, utilising multi-mode approaches such as postal surveys and follow-ups can increase the response rate to 60% plus [26]. Based on these findings and the study sample, a response rate of 40% was deemed satisfactory. Although the survey achieved this target, individual questions had a response rate varying from 24 to 100%. Improved response rate to the questions may have been achieved by other qualitative methods such as interviews and focus groups. However, for reasons already stated, this will not feasible.

As a qualitative methodology employing a quantitative data collection method, a number of techniques were employed to assess the rigour of the study. Face and content validity were evaluated through the use of a pilot study of experts [27]. With no other questionnaires measuring the current manufacturing and fitting of artificial eyes, the questionnaire items were taken from the literature review and service visits ensuring they were practically and theoretically relevant to the constructs being studied. This method of triangulation provides credibility to the study. Consistency throughout the research process, specifically in conceptualising the study, collecting the data, interpreting the findings, reporting the results and referencing the literature ensures dependability and confirmability. As a cross-sectional design, this study provides a snapshot of how things are for respondents at the time it was administered. However, the phenomenon will not remain stagnant, affecting its transferability. By reporting clear descriptions of the phenomena being studied and the context in which the study took place, the person wishing to ‘transfer’ the results can judge its suitability.

### Implications

The findings of this study have implications for both the development of artificial eyes and for future research. First the results indicate the importance of the child’s patient’s psychological wellbeing in enabling the smooth running of the current process where a good fit and an aesthetically pleasing artificial eye can be achieved. Factors that affect the child’s adjustment require investigation. This will firstly come from understanding the impact that the process and its associated factors have on the child from their perspective. Specific factors that were highlighted in this study include the influence of the parent’s, the ocularist and wider society upon the child patient’s adjustment and the effect specific techniques have on the patient.

Secondly, the study highlighted a mixed reaction to the incorporation of technology into the process. Although it may be met with resistance, investigation is needed to ascertain whether and how it can assist, and if not, what adjustments are needed to make it possible. Benefits of utilising current technology include a better process and outcome which consequently can enhance the psychological wellbeing of the patient, thus, improve their quality of life.

Finally, it was highlighted by some respondents that there is a need for recognition and certification of those working in the field. Often seen as part of a larger prosthetic team, funding opportunities that focus specifically on artificial eye development are few and far in between. Consequently, any development of the process is down to ocularists, based on their experience and using their time, equipment and materials. By investing in the field, research and development can be undertaken on a larger scale.

## Conclusion

This qualitative study employing a questionnaire method and a mixed data analysis approach has shown that many factors play a role in the manufacturing and fitting of artificial eyes in children diagnosed with retinoblastoma. The process can be seen as an interaction rather than linear in nature as its success is dependent on the child patient’s acceptance and adjustment which is dependent on the factors associated to the process. Therefore, investigation into how all aspects of the process can be improved will be beneficial for those involved.
